# Detection and Quantitation of Adulterated Paprika Samples Using Second-Order HPLC-FLD Fingerprints and Chemometrics

**DOI:** 10.3390/foods11152376

**Published:** 2022-08-08

**Authors:** Xiaodong Sun, Min Zhang, Pengjiao Wang, Junhua Chen, Shengjun Yang, Peng Luo, Xiuli Gao

**Affiliations:** 1 State Key Laboratory of Functions and Applications of Medicinal Plants, School of Pharmacy, Guizhou Medical University, Guiyang 550025, China; 2 Microbiology and Biochemical Pharmaceutical Engineering Research Center of Guizhou Provincial Department of Education, Guizhou Medical University, Guiyang 550004, China; 3 Guizhou Provincial Engineering Research Center of Food Nutrition and Health, School of Public Health, Guizhou Medical University, Guiyang 550025, China

**Keywords:** paprika, HPLC-FLD, chemometrics, second-order fingerprint, food authentication

## Abstract

Paprika is a widely consumed spice in the world and its authentication has gained interest considering the increase in adulteration cases in recent years. In this study, second-order fingerprints acquired by liquid chromatography with fluorescence detection (HPLC-FLD) were first used to detect and quantify adulteration levels of Chinese paprika samples. Six different adulteration cases, involving paprika production region, cultivar, or both, were investigated by pairs. Two strategies were employed to reduce the data matrices: (1) chromatographic fingerprints collected at specific wavelengths and (2) fusion of the mean data profiles in both spectral and time dimensions. Afterward, the fingerprint data with different data orders were analyzed using partial least squares (PLS) and n-way partial least squares (N-PLS) regression models, respectively. For most adulteration cases, N-PLS based on second-order fingerprints provided the overall best quantitation results with cross-validation and prediction errors lower than 2.27% and 20.28%, respectively, for external validation sets with 15–85% adulteration levels. To conclude, second-order HPLC-FLD fingerprints coupled with chemometrics can be a promising screening technique to assess paprika quality and authenticity in the control and prevention of food frauds.

## 1. Introduction

Paprika, the dehydrated and milled fruit of certain varieties of red pepper (*Capsicum annuum* L.), is one of the most widely consumed spices in many areas of the food industry [1,2]. Owing to its particular taste (sweet and spicy), flavor, and high coloring capacity, paprika can be used as a food additive, acting as both a natural colorant and a flavoring agent [3]. In China, paprika is widely used in a wide variety of cooking methods for both flavor and color [4]. Moreover, it is also well-known to be a good source of micronutrients (minerals and vitamins) and bioactive compounds such as capsaicinoids, carotenoids, and phenolic and polyphenolic compounds [5]. These compounds exert multiple pharmacological and physiological effects including analgesic, anti-obesity, antioxidant, cardioprotective, and anticancer activities [6,7,8].

The characteristics of paprika can be affected by cultivar, geographical region, agronomic conditions, and product process. Hence, paprika from different regions and cultivars may differ in quality and composition. This also indicates that the quality of paprika, which is mainly reflected by its prices and commercial values, may also differ within the same cultivar. With the increasing consumption of paprika worldwide, it has become an attractive target for adulteration and mislabeling. Generally, high-quality paprika, such as protected geographical indication (PGI) paprika products, can be subjected to adulteration more easily due to its high reputation and commercial values. Paprika adulteration can be performed in various forms and may occur at each stage of production in different ways: addition of foreign matter, addition of inferior products or cheaper materials, and adulteration by masking poor quality with colorants [9]. Adulterants are usually incorporated at low levels and are difficult to detect by a simple sensory evaluation due to their similar color, appearance, and texture to the real paprika samples [10,11]. Therefore, accurate and reliable analytical strategies to detect and prevent paprika frauds are demanded.

Due to the complexity of compound distribution in foods and the lack of specific compounds directly related to their origin or quality, classic targeted analysis may not be competent for food authentication issues. Hence, the use of nontargeted approaches, by recording instrumental signals associated with large numbers of known and unknown compounds present in the samples (fingerprinting approaches), has been increasing in recent years [12]. Regarding paprika, the application of chemometrics to the nontargeted instrumental data acquired using several spectroscopic techniques, such as UV-Vis [13,14], fluorescence [15], Raman [16], visible-Near-infrared (VIS-NIRS) [17], and infrared spectroscopies [3], for the authentication of adulterated paprika has been attempted. Moreover, nontargeted fingerprints obtained by liquid chromatography techniques have also played an important role in the authentication or discrimination of paprika. To date, liquid chromatography coupled with different detection systems, such as ultraviolet (LC-UV) [7], fluorescence (LC-FLD) [18], electrochemical detection (LC-ECD) [19], and mass spectrometry (LC-MS) [20], has been evaluated.

However, fingerprinting approaches based on less expensive chromatographic instrumentals (e.g., LC-UV and LC-FLD) usually only record signals at predefined UV absorption or excitation/emission wavelengths [7,18]. Despite the potentiality of first-order chromatographic data as chemical descriptors to address the paprika origin and cultivar having been demonstrated, chemical information provided by HPLC can be partially lost or corrupted and may be unsuitable for paprika authentication issues sometimes. HPLC coupled with diode array detection (DAD) or fluorescence detection (FLD) allows the acquisition of information (second-order data) on the retention behavior and structure-related spectral patterns of the solutes [21], providing more chemical information on the paprika fingerprints as compared to that obtained by a univariate chromatogram. Therefore, it is expected to further improve the performance of paprika authentication by applying the chromatographic fingerprinting approach that combines second-order data with chemometrics. As far as we know, no studies have been reported for the detection or authentication of paprika using comprehensive information carried by HPLC-DAD or FLD spectrochromatogram and chemometrics.

Driven by the above-mentioned problems and inspired by the abundant information of second-order data, nontargeted second-order HPLC-FLD fingerprints combined with chemometrics were first used to detect and quantify adulteration levels in fraudulent paprika samples, involving production region, cultivar adulteration, and both. On the one hand, the second-order fingerprint data, strongly related to phenolic acid and polyphenolic compounds, were used directly to develop regression models using the n-way partial least squares (N-PLS) algorithm. On the other hand, two variable reduction strategies were applied to reduce the data matrices for each sample, and then the reduced data (first-order fingerprints) were analyzed by partial least squares (PLS). To fully demonstrate the superiority of second-order fingerprint data in the detection of adulterated paprika, quantitation results obtained from N-PLS and PLS were investigated and compared meticulously. The flow chart of the whole experimental steps is shown in Figure S1 in the Supplementary Materials.

The objective of this work is not only to detect and quantify adulteration levels in fraudulent paprika samples, but also to explore the data pretreatments, data orders, and chemometric methods of nontargeted fingerprints based on HPLC-FLD to improve quantification performance. To achieve this goal, different methods (PLS and N-PLS) for the detection and quantification of adulterated paprika samples using first-order and second-order HPLC-FLD fingerprints are discussed and compared.

## 2. Materials and Methods

### 2.1. Reagents and Materials

HPLC-grade methanol and acetonitrile were purchased from Merck (Darmstadt, Germany). Formic acid (≥99%) was obtained from Macklin (Shanghai, China). HPLC-grade water was supplied by Wahaha (Hangzhou, China).

### 2.2. Samples

A total of 80 paprika samples from different regions and cultivars were collected in May 2021. Among the samples of each region, there were different cultivars of paprika: Erjingtiao (EJT), Lantern (LT), Bullet (BL), Xiaomi (XM), Wanzi (WZ), and Pod (PD) in the samples from Guizhou; Bullet and Neihuang New Generation (NH) in the samples from Henan; Erjingtiao in the samples from Sichuan. The characteristics of these paprika samples, including pungency degree, flavor, and main origin, are collected in Table S1. For example, NH pepper is a variant of Pod pepper and is mainly produced in Henan, which has a high flavor and pungency degree. This also suggests that NH paprika may contain more capsaicinoids and characteristic flavor compounds than others. Moreover, the number of samples for each type of paprika is summarized in Table S2. All the samples were obtained directly from paprika producers or local markets.

Six paprika adulteration cases involving different production regions and cultivars were studied: Sichuan EJT adulterated with Guizhou EJT (case 1), Guizhou LT adulterated with Henan LT (case 2), Guizhou LT adulterated with Guizhou EJT (case 3), Guizhou XM adulterated with Henan NH (case 4), Guizhou BL adulterated with Henan BL (case 5), and Guizhou WZ adulterated with Guizhou PD (case 6). Binary paprika samples were prepared by mixing different types of paprika (region and cultivar) with different proportions. The selection of real samples and adulterated samples was mainly based on the prices and commercial values of different paprika samples. For example, the price of Sichuan EJT is higher than that of Guizhou EJT, which makes the former more likely to be adulterated. To achieve the detection of adulterated samples and quantitation of their adulterant levels by chemometrics, calibration and external validation blends were prepared for each case as shown in Table 1. The 100% pure and adulterated paprika samples in the calibration set were 10 and 6 replicates, while others were 4 replicates. Moreover, two extra quality control (QC) samples with 50% adulteration levels were prepared to evaluate the repeatability and robustness of the chemometric results as well as to detect systematic errors. Hence, a total of 54 samples can be obtained in each adulteration case studied.

A sample pretreatment procedure based on solid-liquid extraction (SLE) using water: acetonitrile (20:80 *v*/*v*) was performed [22]. Briefly, 0.3 g of paprika was weighed and mixed with a 3 mL solution and then vortexed for 1 min. Subsequently, samples were sonicated for 20 min and centrifuged at 3000 rpm for 15 min. The supernatant was diluted 10-fold with the same solution and filtered through a 0.45 μm nylon filter before being stored at 4 °C before use.

### 2.3. Instrumentation

The HPLC analyses were performed using a Dionex Ultimate 3000 HPLC system (Thermo Scientific, Germering, Germany) equipped with an Ultimate 3000 pump, an Ultimate autosampler, an Ultimate column compartment, and a fluorescence detector (FLD). The Chromeleon software 7.2.10 (Thermo Scientific) was used to control the HPLC system and data acquisition.

The chromatographic separations were achieved using a Dikma C18 reverse-phase column (100 mm × 4.6 mm i.d., 5 μm particle size), with a guard column packed with the same material. The column was maintained at 35 °C throughout the analysis. The mobile phase was 0.1% formic acid aqueous solution (A) and acetonitrile (B), delivered at a flow rate of 1.0 mL/min. The gradient elution procedure was set as follows: 2.0 min, 20% B; 4.0 min, 90% B; 10.0 min, 90% B; 11.0 min, 20% B; 16.5 min, 20% B. The injection volume was 10 μL. Moreover, the HPLC-FLD fingerprints (data matrices for samples) were acquired at 310 nm for excitation wavelengths and 350 nm to 450 nm for emission wavelengths. The temperature of the flow cell was 45 °C, while the sensitivity of the FLD detector was set to 5.

### 2.4. Chemometrics

The raw HPLC-FLD fingerprints were processed in the MATLAB environment (version R2010b) after converting them to text files. A data matrix of size 2250 × 101 (elution time points × spectroscopic data points) was obtained for each analytical sample. As the fingerprint data were reproducible between successive runs, there was no need to perform any chromatographic alignment procedure before chemometric analysis. All the HPLC-FLD fingerprints were autoscaled before modeling to provide the same weight to each variable.

Herein, PLS was performed using regression toolbox 1.3 [23], while PLS-DA was used by classification toolbox 5.4 [24]. N-PLS analyses were applied using the N-way toolbox, which is freely available at http://www.models.kvl.dk/algorithms (accessed on 10 August 2021) [25]. All the interface graphics and toolboxes were designed for MATLAB software. Briefly, the X block in N-PLS is a three-way data array **X** (second-order fingerprints) with the size of *I* (samples) × *J* (retention time points) × *K* (emission wavelengths), while those in PLS and PLS-DA are matrixes (first-order fingerprints) with the size of *I* (samples) × *J* (variables). In contrast, the Y block is a data matrix that defines each adulterant percentage in PLS and N-PLS, while defining each sample class in PLS-DA. More detailed information and theoretical descriptions of these chemometric algorithms can be found in the relevant literature [24,26].

To evaluate the analytical performances of different chemometric methods/fingerprints, a series of model parameters including linearity (R^2^), root-mean-square error of calibration (RMSEC), root-mean-square error of cross-validation (RMSECV), and root-mean-square error of validation (RMSEV) were calculated and compared. Moreover, the optimum number of latent variables (LVs) of PLS, N-PLS, and PLS-DA was chosen at the first significant minimum point of the cross-validation (CV) error by a 10-fold Venetian blind approach.

## 3. Results and Discussion

### 3.1. General Concerns

In recent years, FLD has become a feasible alternative and also a complement to UV and MS detection due to its high selectivity and sensitivity. Generally, FLD can provide better detection capabilities for fluorescent compounds than UV detection, even comparable to that of MS detection [27]. As some food extracts contain a lot of fluorescent components related to their origin, HPLC-FLD fingerprinting has gained wide concern in food authentication. Thus far, HPLC-FLD fingerprints combined with chemometrics have been successfully used for addressing the classification and authentication of food samples, such as paprika, coffee, tea, extra-virgin olive oil, and nuts [12,18,28,29,30,31,32]. Moreover, in the cases of nuts classification and coffee classification, HPLC-FLD provided better discrimination performances than HPLC-UV [29,30].

In a recent study [18], the applicability of nontargeted HPLC-FLD fingerprints (first-order data) related to phenolic and polyphenolic compounds has been demonstrated for the authentication of paprika samples according to their production regions and types. Inspired by this, we aimed to further explore the potential of second-order HPLC-FLD fingerprint data in the detection and quantitation of adulterated paprika samples from different provinces of China. Herein, nontargeted second-order fingerprints were obtained using HPLC-FLD by setting the excitation wavelength at 310 nm and the emission wavelengths from 350 to 450 nm. The selection of excitation and emission wavelengths was based on the published literature [18] and our preliminary experiments. The results showed that second-order fingerprints recorded at the excitation wavelength of 310 nm and emission wavelengths of 350 nm to 450 nm had better overall intensities. These fingerprints are based on the instrumental response (data matrix) recorded as a function of elution time and emission wavelength, roughly related to bioactive compounds (e.g., phenolic and polyphenolic compounds) in paprika samples [18].

**Figure 1:**
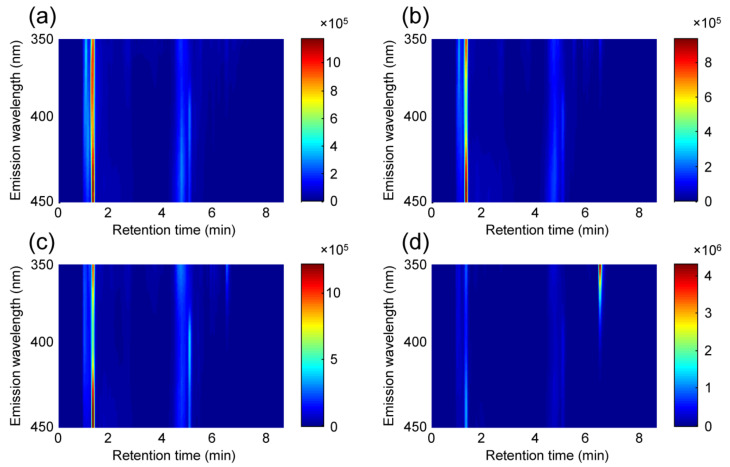
Counter maps of second-order HPLC-FLD fingerprints of (**a**) Sichuan EJT, (**b**) Guizhou EJT, (**c**) Guizhou BL, and (**d**) Henan BL after subtracting the solvent blank.

Given the characteristics of nontargeted analysis, the optimization of chromatographic condition was based on obtaining enough discriminative information in a suitable time, rather than seeking baseline-resolved peaks. Accordingly, a rapid gradient elution procedure, using a short C18 column and 0.1% formic acid and acetonitrile, was used for HPLC-FLD data acquisition. Figure 1 depicts the counter maps of fingerprints of four representative paprika samples after a simple blank subtraction procedure. As shown, similar fingerprints were obtained for different kinds of paprika samples, roughly reflected in the number of detectable peaks and the overall chromatographic-spectral profiles. The main differences in these fingerprints, derived from their fluorescence intensities, provided the possibility to quantify the paprika adulterant levels by chemometric methods. Moreover, only the fingerprint data recorded at 0 to 8.7 min were considered, avoiding the column re-equilibration step.

### 3.2. Variable Reduction

**Figure 2:**
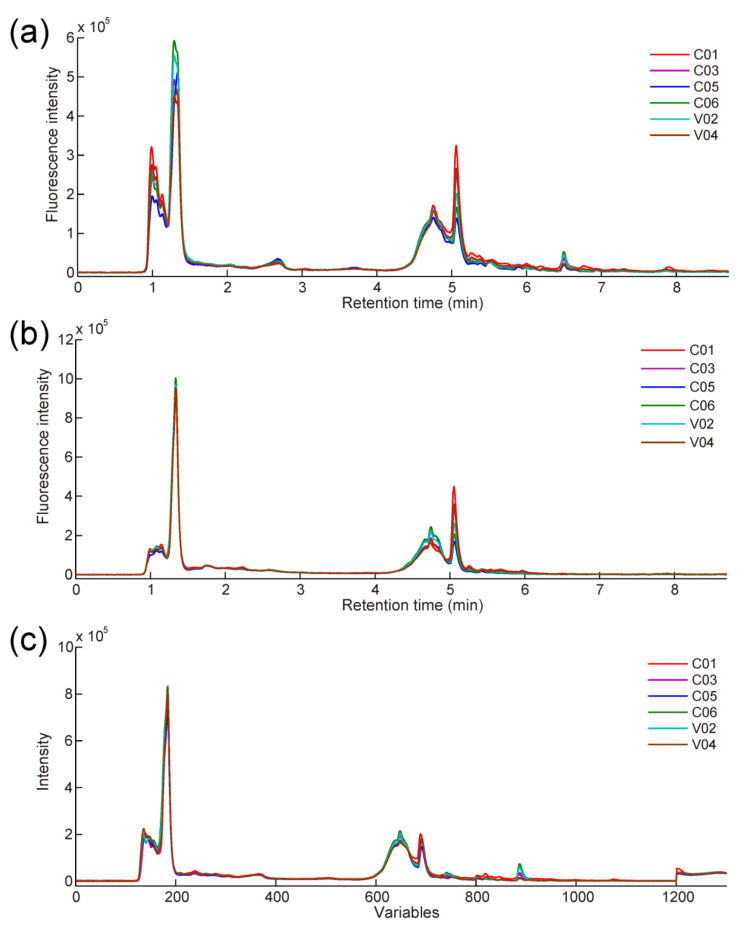
First-order fingerprint signals of selected samples from case 6 (Guizhou WZ adulterated with Guizhou PD) collected at 380 nm (**a**) and 440 nm (**b**), respectively; first-order fusion fingerprint signals of selected samples from case 4 (Guizhou XM adulterated with Henan NH) generated by DVF (**c**).

As there were fewer chemometric algorithms directly using second-order data for modeling, two variable reduction strategies were employed to obtain first-order fingerprint data suitable for conventional chemometric methods such as PLS and PLS-DA. The first one selected HPLC-FLD fingerprints collected at specific detection emission wavelengths (380 and 440 nm), which were picked through the literature reported [18] and through visual inspection of Figure 1. Two data matrices with the size of 54 × 1200 (samples × variables) were obtained for each adulteration case by this way. The second used decomposition and vector fusion (DVF), a new variable reduction strategy developed by Jiménez-Carvelo [33], to generate “chromatographic + spectral” fusion fingerprint data. Briefly, a first-order vector consisting of 1301 variables could be obtained by fusing the retention time mean vector (1200 × 1) and emission spectral vector (101 × 1) of each sample. Then, the fused vectors of all samples were grouped in a single matrix of size 54 × 1301 (samples × variables). Compared with first-order fingerprints obtained at specific detection wavelengths, fingerprints generated by DVF retained more sample information, which might be more beneficial for the detection and quantification of adulterated paprika samples. Figure 2a,b display the signals of samples from one adulteration case (Guizhou WZ adulterated with PD) collected at 380 nm and 440 nm, respectively. Figure 2c presents the fused mean vectors of samples from another adulteration case (Guizhou XM adulterated with Henan NH).

### 3.3. Detection and Quantitation of Adulteration by PLS

**Figure 3:**
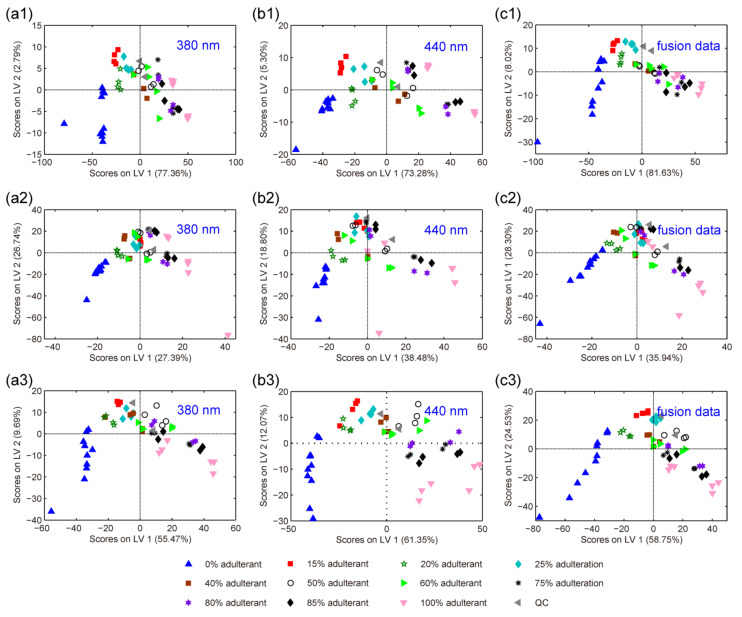
PLS-DA score plots of LV1 versus LV2, using first-order fingerprint data collected at 380 nm (**a1**–**a3**), first-order fingerprint data collected at 440 nm (**b1**–**b3**), and first-order fusion fingerprint data generated by DVF (**c1**–**c3**) in Sichuan EJT adulterated with Guizhou EJT (case 1), Guizhou LT adulterated with Henan LT (case 2), and Guizhou WZ adulterated with Guizhou PD (case 6), respectively.

The feasibility of conventional first-order HPLC-FLD fingerprints as chemical descriptors to detect and quantify paprika adulterations by PLS regression was investigated in the six adulteration cases. First, three kinds of first-order fingerprints acquired by variable reduction strategies were analyzed by PLS-DA to observe the distribution of samples from a two-dimensional space for both calibration and validation sets. The score plots of PLS-DA for three of the studied adulteration cases, Sichuan EJT adulterated with Guizhou EJT, Guizhou LT adulterated with Henan LT, and Guizhou WZ adulterated with Guizhou PD, are illustrated in Figure 3. As can be seen, although different first-order fingerprints may reveal different feature information, samples tend to be distributed along the first score (LV1) according to their adulteration levels. For example, the real paprika samples (0% adulterant) are located at the left of the score plot, while the 100% pure adulterant samples are at the right. The distribution of samples in the score plots reveals differences in their production region and cultivar. Moreover, two QC samples are usually clustered in the center of the score plots, which demonstrated the robustness of the PLS-DA analysis and the feasibility of conventional first-order HPLC-FLD fingerprints.

Then, PLS regression models were established using first-order fingerprint data from the calibration sets, and their quantitation performances were evaluated by analyzing binary mixed samples with different adulteration levels (validation sets) not involved in modeling. Figures S2 and S3 present the plots of predicted adulteration levels versus real levels obtained by PLS regression in six cases. As can be seen, although it seemed to build good calibration models for three types of first-order fingerprints by PLS, with all RMSEC values below 4.97% and R^2^ ≥ 0.983, poor predicted results were obtained for validation sets in some cases. For example, scatter points of the validation set significantly deviated from the established PLS regression curves in case 3, resulting in all RMSEV values exceeding 20% (Figure S2). Relatively poor predicted results were also obtained in cases 2 and 4. For these cases, one could find that errors were mainly from validation samples with low adulteration levels—all PLS regression models significantly overestimated them. Moreover, no considerable improvement was observed in the predicted results by PLS using first-order fusion data (c in Figures S2 and S3), as compared to data collected at specific wavelengths (a,b in Figures S2 and S3). Hence, PLS regression models constructed using first-order data were insufficient to achieve satisfactory quantitation results in all adulteration cases studied. This might be because such data contained less comprehensive information or experimental variance that was relevant to the quantitation, thus demoting the model prediction performance in some cases to a large extent.

### 3.4. Detection and Quantitation of Adulteration by N-PLS

**Figure 4:**
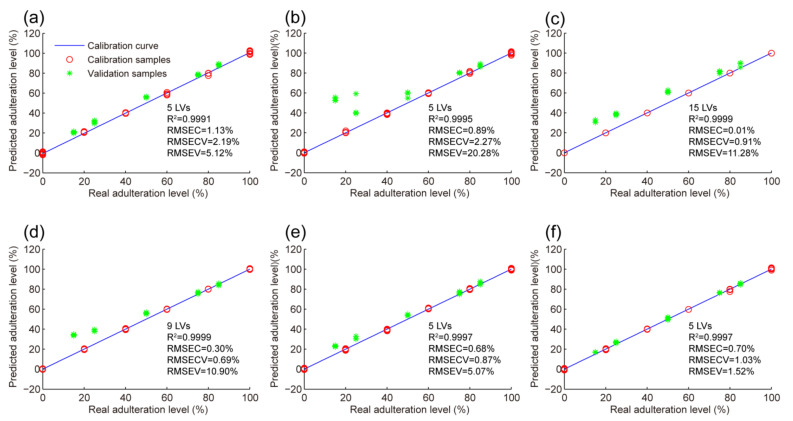
The plots of predicted adulteration levels versus real values obtained by N-PLS regression for six adulteration cases (**a**–**f**).

Considering the more abundant and comprehensive information provided by second-order fingerprints, an attempt was made to detect and quantify adulteration levels of paprika by N-PLS. The quantitation results obtained by N-PLS for six adulteration cases are shown in Figure 4 and Table 2. As can be seen, overall good results were obtained in almost all cases studied (except case 2), with a linearity (R^2^) higher than 0.999 and RMSEC and RMSEV values below 1.13% and 11.28%, respectively. Moreover, the regression curves established by N-PLS provided overall good predictions for samples with low adulteration levels. In addition, although the prediction error (RMSEV = 20.28%) in case 2 was still large, significant improvements in prediction results could be observed in other cases when comparing the results of N-PLS with those obtained by PLS. For instance, the RMSEV in case 3 decreased from approximately 20% to 10% when using PLS and N-PLS, respectively. Given the fact that more accurate results can be obtained, second-order fingerprint data in combination with the N-PLS algorithm are more suitable for quantifying adulteration levels in paprika samples.

In addition, when comparing the results with those previously reported by first-order HPLC-FLD fingerprints [18], a certain improvement was observed. While similar prediction errors were obtained with both first-order and second-order fingerprints, calibration errors obtained by the latter were generally lower, with most RMSEC (cases 3, 4, 5, and 6) and RMSECV (cases 3, 4, 5, and 6) values below 0.70% and 1.03%, respectively. Although the experiment procedures and samples employed were different between these two methods, analytical results also indicated that the second-order fingerprints, which contained more information, might have advantages in the detection and quantification of adulterated paprika samples to some extent.

## 4. Conclusions

In this work, nontargeted second-order HPLC-FLD fingerprints coupled with chemometrics were successfully used for the detection and quantitation of adulteration levels in fraudulent paprika samples. Six different adulteration cases, involving paprika production region, cultivar, or both, were investigated by pairs. Two variable reduction strategies were employed to reduce the data matrices and generate first-order data for samples. To better detect adulterated paprika samples and evaluate the potential of fingerprints with different data orders in chemometric analysis, PLS and N-PLS were used to establish regression models using first-order and second-order fingerprint data, respectively. No obvious differences in the quantitation performances were found by PLS models when using first-order fusion data and first-order data collected at specific wavelengths. However, although N-PLS and PLS shared similar calibration errors (RMSEC) for almost all cases, more satisfactory predicted results with lower RMSEV were obtained for the former one, especially in cases 2 and 4. Moreover, when compared with the results previously published using first-order HPLC-FLD fingerprints, a certain improvement of results with generally lower calibration errors was obtained based on second-order HPLC-FLD fingerprints.

To conclude, the superiority of second-order HPLC-FLD fingerprint data to detect and quantify adulterated paprika has been proved in this work. The quantitation results obtained from N-PLS using second-order data were more satisfactory than those obtained from PLS using first-order data. This improvement is probably due to second-order data capturing more abundant chemical information (features or descriptors) that facilitates quantification. Therefore, nontargeted second-order HPLC-FLD fingerprints in combination with chemometrics can be a useful screening technique to assess paprika integrity and authenticity in the control and prevention of food frauds.

## Figures and Tables

**Table 1:** Concentration levels designed for paprika adulteration cases in calibration and validation sets, where X is the pure paprika samples and Y is the adulterated ones.

	Calibration Set						Validation Set				
	C01	C02	C03	C04	C05	C06	V01	V02	V03	V04	V05
X (%)	100	80	60	40	20	0	15	25	50	75	85
Y (%)	0	20	40	60	80	100	85	75	50	25	15
replicates	10	4	4	4	4	6	4	4	4	4	4

**Table 2:** Results for the quantitation of adulteration levels in six cases using second-order HPLC-FLD fingerprints and N-PLS.

Original Paprika	Paprika Used as Adulterant	LV	Linearity (R^2^)	RMSEC (%)	RMSECV (%)	RMSEV (%)
Sichuan EJT	Guizhou EJT	5	0.9991	1.13	2.19	5.12
Guizhou LT	Henan LT	5	0.9995	0.89	2.27	20.28
Guizhou LT	Guizhou EJT	15	0.9999	0.01	0.91	11.28
Guizhou XM	Henan NH	9	0.9999	0.30	0.69	10.90
Guizhou BT	Henan BT	5	0.9997	0.68	0.87	5.07
Guizhou WZ	Guizhou PD	5	0.9997	0.70	1.03	1.52

## Data Availability

The data presented in this study are available on request from the corresponding author.

## Supplementary Materials

The following supporting information can be downloaded at: https://www.mdpi.com/article/10.3390/foods11152376/s1, Figure S1: The flow chart of the whole experimental steps; Figure S2. The plots of predicted adulteration levels versus real values obtained by PLS regression using first-order data collected at 380 nm (a1–a3), 440 nm (b1–b3) and first-order fusion data (c1–c3) in case1 (a1–c1), case 2 (a2–c2) and case 3 (a3–c3), respectively; Figure S3. The plots of predicted adulteration levels versus real values obtained by PLS regression using first-order data collected at 380 nm (a1–a3), 440 nm (b1–b3) and first-order fusion data (c1–c3) in case4 (a1–c1), case 5 (a2–c2) and case 6 (a3–c3), respectively; Table S1. The pungency degree, flavor and main origin of paprika samples studied; Table S2. Detailed sample information of the paprika samples analyzed in the study.
