# Supplementary material for: Detection and Quantitation of Adulterated Paprika Samples Using Second-Order HPLC-FLD Fingerprints and Chemometrics
Source: Foods. 2022 Aug 8;11(15):2376. doi: 10.3390/foods11152376 (PMC9368040; doi:10.3390/foods11152376)
Supplement: Supplementary file 1 [file foods-11-02376-s001.zip › foods-1818639-SI.pdf]

foods

Supplementary Materials

Detection and Quantitation of Adulterated Paprika Samples Using Second-Order HPLC–FLD Fingerprints and Chemometrics

Xiaodong Sun [1,2], Min Zhang [1,2], Pengjiao Wang [1,2], Junhua Chen [1,2], Shengjun Yang [1,2], Peng Luo [3] and Xiuli Gao [1,2,3,*]

[1] State Key Laboratory of Functions and Applications of Medicinal Plants, School of Pharmacy, Guizhou Medical University, Guiyang 550025, China; sunxiaodong@gmc.edu.cn (X.-D.S.); minzhang@gmc.edu.cn (M.Z.); wangpengjiao@gmc.edu.cn (P.-J.W.); chenjh@gmc.edu.cn (J.-H.C.); yangshengjun@gmc.edu.cn (S.-J.Y.)
[2] Microbiology and Biochemical Pharmaceutical Engineering Research Center of Guizhou Provincial Department of Education, Guizhou Medical University, Guiyang 550004, China
[3] Guizhou Provincial Engineering Research Center of Food Nutrition and Health, School of Public Health, Guizhou Medical University, Guiyang 550025, China; luopeng@gmc.edu.cn
* Correspondence: gaoxl@gmc.edu.cn.

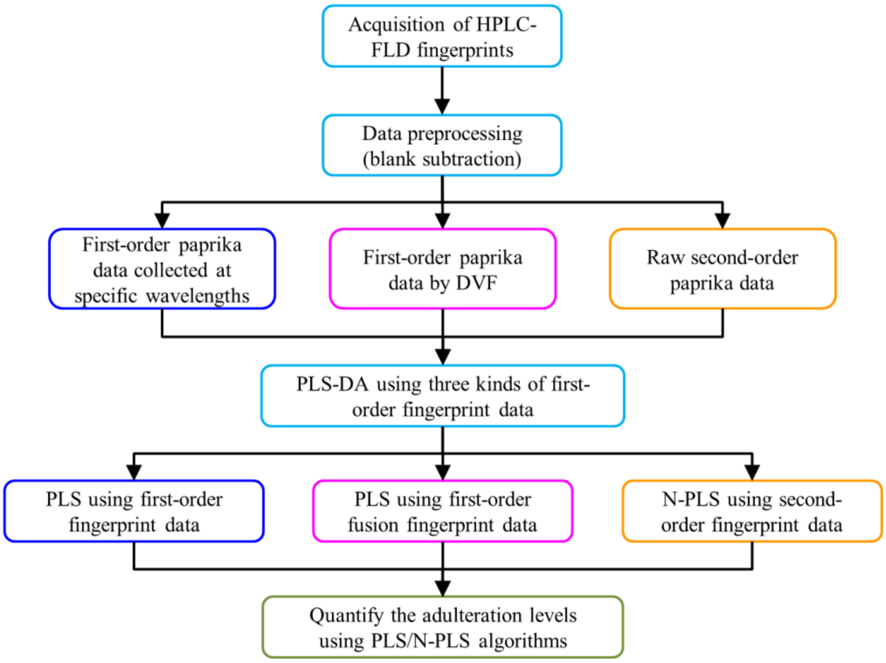

Figure S1. The flow chart of the whole experimental steps.

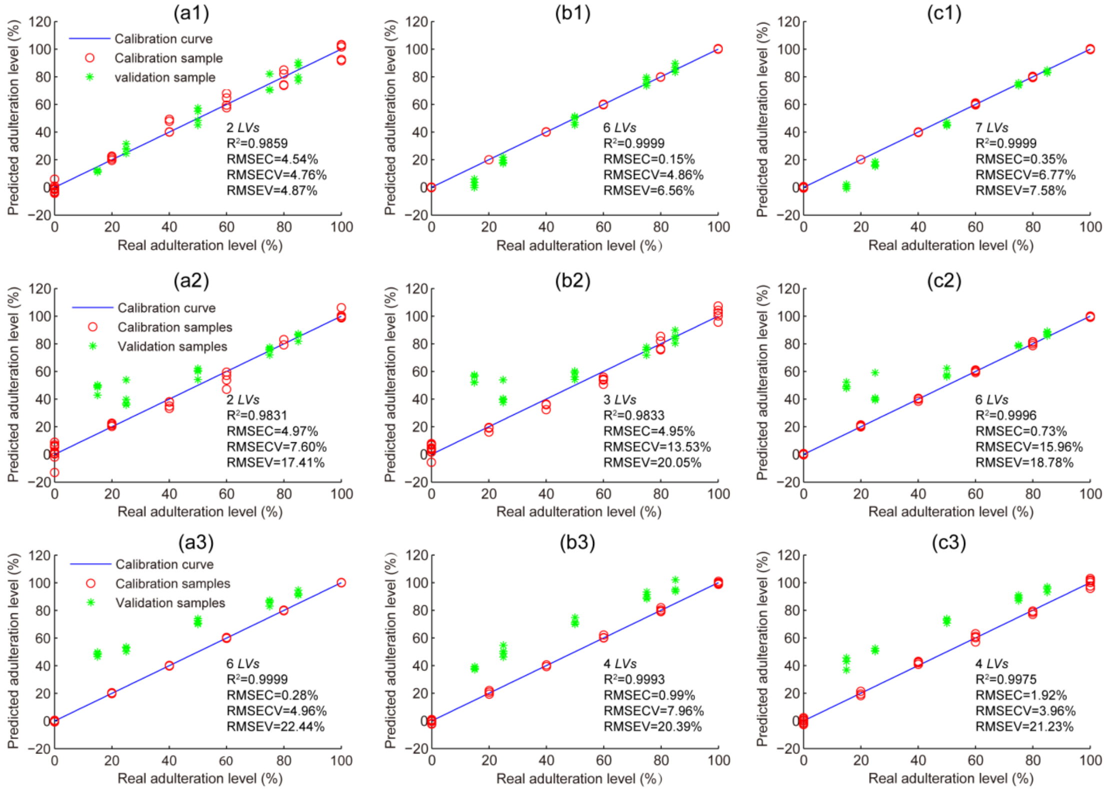

Figure S2. The plots of predicted adulteration levels versus real values obtained by PLS regression using first-order data collected at 380 nm (a1-a3), 440 nm (b1-b3) and first-order fusion data (c1-c3) in case1 (a1-c1), case 2 (a2-c2) and case 3 (a3-c3), respectively.

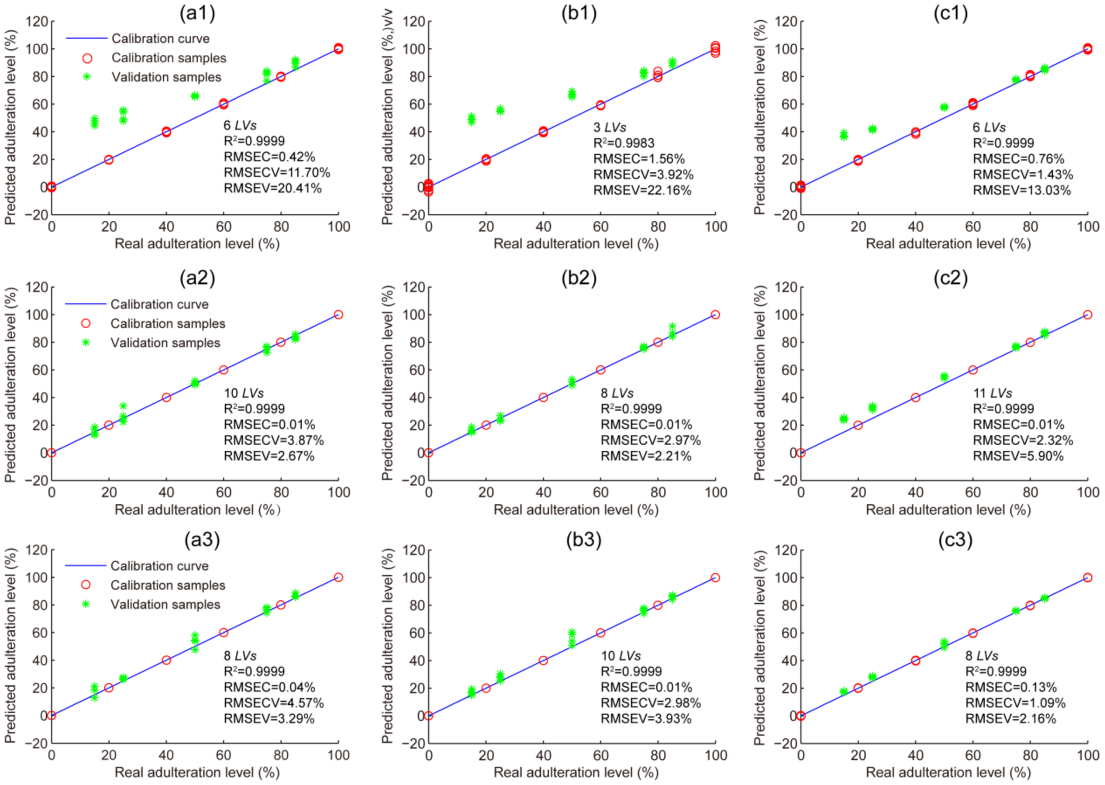

Figure S3. The plots of predicted adulteration levels versus real values obtained by PLS regression using first-order data collected at 380 nm (a1-a3), 440 nm (b1-b3) and first-order fusion data (c1-c3) in case4 (a1-c1), case 5 (a2-c2) and case 6 (a3-c3), respectively.

Table S1. The pungency degree, flavor and main origin of paprika samples studied.

Variety	Pungency degree	Flavor	Main origin
Erjingtiao	★★★	★★★★★	Sichuan, Guizhou, Henan
Lantern	★★★	★★★★★	Most provinces in China
Bullet	★★★★	★★★★	Sichuan, Guizhou, Henan
Xiaomi	★★★★★	★★★	Hunan, Yunnan, Guizhou, Sichuan
Pod	★★★	★★★★	Most provinces in China
Wanzi	★★★	★★★★	Jinsha County, Guizhou
Neihuang New Generation	★★★★★	★★★★★	Henan

Table S2. Detailed sample information of the paprika samples analyzed in the study.

Variety	Origin region	Number of samples
Erjingtiao	Sichuan	10
Lantern	Guizhou	10
Xiaomi	Guizhou	10
Bullet	Guizhou	10
Wanzi	Guizhou	10
Erjingtiao	Guizhou	6
Lantern	Henan	6
Neihuang New Generation	Henan	6
Bullet	Henan	6
Pod	Guizhou	6
**Total**		**80**
